# Enhancement of tribological performance of lubricants using polydimethylsiloxane powder additives

**DOI:** 10.1039/d4ra05164e

**Published:** 2024-09-30

**Authors:** Sung-Jun Lee, Dawit Zenebe Segu, Chang-Lae Kim

**Affiliations:** a Department of Mechanical Engineering, Chosun University Gwangju 61452 Republic of Korea kimcl@chosun.ac.kr

## Abstract

This study explored the potential enhancement of lubrication performance by incorporating polydimethylsiloxane (PDMS) powder as a lubricant additive. The PDMS powder was successfully synthesized *via* mechanical and thermal processes, exhibiting a particle size distribution with an average diameter of 39 μm. XRD and FTIR analyses confirmed the amorphous structure and chemical composition of the PDMS-based silicone rubber powder. XPS analysis provided evidence of increased crosslinking in the crosslinked PDMS powders, with shifts in binding energies and changes in elemental ratios, supporting enhanced Si–O–Si network formation. The addition of the powder to the Poly alpha Olefin (PAO)-based lubricating oil induced notable changes in the physicochemical properties, including viscosity and contact angle. Friction tests revealed that the introduction of silicone rubber powder did not compromise the friction behavior of the lubricating oil owing to the soft and deformable nature of the powder particles, which minimized direct metal-to-metal contact. A significant observation was the decreasing trend in the wear rate with increasing powder concentration, which reached a minimum at 2 wt%. This phenomenon is attributed to a synergistic combination of particle deformation, stress absorption/dispersion effects, and lubricating film formation. Optical microscopy analysis provided visual evidence supporting the low wear rate, with specimens containing 1 wt% and 2 wt% concentrations exhibiting narrow wear widths and reduced wear particle adhesion. Further augmentation of the wear resistance and durability is anticipated through the addition of silicone rubber curing agents, which are expected to bolster the mechanical strength and load-bearing capacity of PDMS powder particles while enhancing the stability of the lubricating film.

## Abstract 2

This study explored the potential enhancement of lubrication performance by incorporating polydimethylsiloxane (PDMS) powder as a lubricant additive.

## Graphical abstract

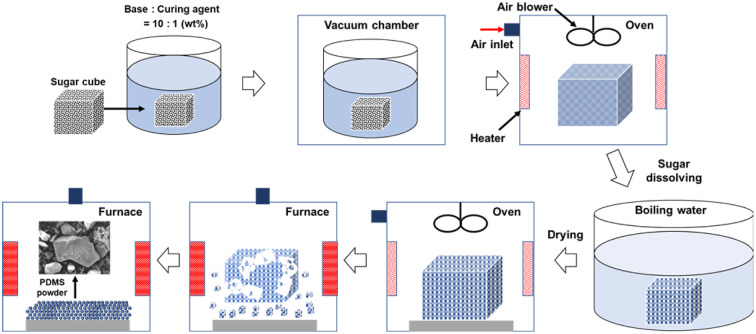

## 1 Introduction

Efficient and sustainable lubrication in mechanical systems has long been a focal point in the mechanical industry and engineering.^1,2^ While metal particles or nanoparticles have been utilized as additives to enhance lubrication performance, their superior lubricity is often accompanied by accelerated wear of interacting surfaces over prolonged usage periods.^3,4^ Additives forming lubricating films in lubricants induce the wear of interacting mechanical components because of their high strength upon repetitive contact with machine parts. This issue leads to problems such as reduced efficiency, decreased lifespan of mechanical components, and increased maintenance costs.

Numerous studies have demonstrated the adverse effects of existing high-strength particle-based lubricant additives on the wear. Kong *et al.* conducted research utilizing a reciprocating sliding plate tribometer under boundary lubrication conditions to investigate the influence of graphene particle size and thickness on lubrication performance.^5^ They employed few-layer and multilayer graphene as lubricant additives and Poly alpha Olefin 4 (PAO 4) lubricating oil as the base oil. According to their findings, oils containing multilayer graphene exhibited superior friction/wear reduction compared to pure base oils, which is attributed to the frictional characteristics of graphene with more layers and smaller particles. Nagarajan *et al.* explored the lubrication characteristics of diesel-based engine oil using MoS_2_ and graphene as additives.^6^ The study demonstrated that adding 0.05 wt% of the additive resulted in approximately 58% reduction in friction coefficient and 36% reduction in wear diameter. Del Rio *et al.* evaluated the thermal-physical properties and tribological properties of nanofluids utilizing CaCO_3_ and CeF_3_ nanoparticles as additives.^7^ The performances of the nanofluids were evaluated at mass concentrations of 0.05, 0.1, 0.15, and 0.2 wt%. Although using nanoparticles as lubricant additives reduced the surface tension without affecting the contact angles, a decrease in the friction coefficient was achieved in both types of nanofluids, exhibiting a maximum reduction rate of 13%. Research on the friction and wear properties of high-strength particles as lubricant additives is currently ongoing. However, wear occurs because of the discontinuous contact with materials in contact with high-strength particles. In particular, localized wear, including abrasive wear such as scratch patterns, occurs owing to additives with higher strength than the counterpart materials. To address this issue, researchers have turned their attention to exploring materials that are soft and exhibit excellent lubricity as potential alternatives.^8–10^ Van Ravenstejin *et al.* investigated polymer-based lubricant additives for friction reduction, wear protection, and viscosity modification.^11^ They altered the dependent characteristics of polymer additives on solid surfaces through a combination of polymer topology and organic–inorganic hybrid chemistry. The boundary lubrication performance was evaluated under harsh conditions mimicking automotive applications for 24 h, confirming the performance of additives as lubricants. Such soft materials have emerged as promising solutions for alleviating the wear associated with conventional additives in mechanical components. Although significant advancements have been made in lubrication studies, there is still a critical need to develop additives that can enhance lubrication performance without causing progressive wear of interacting surfaces.

In this study, we propose an innovative approach to enhance lubrication performance by investigating the application of polydimethylsiloxane (PDMS) as a shear lubricant additive. PDMS, widely known as a silicone polymer with characteristics such as low glass transition temperature, high thermal stability, and excellent compatibility, was chosen because of its favorable properties.^12^ PDMS, a silicone-based elastomer, is a promising solution to this challenge. PDMS has been reported to exhibit high friction coefficients owing to its inherently low strength and high adhesiveness under dry conditions; however, it has been reported to possess excellent wear resistance owing to strong bonding in network structures.^13,14^ Additionally, research has been conducted on incorporating PDMS into coatings to reduce friction between contacting surfaces and enhance wear resistance.^15^ These studies have demonstrated that microstructured PDMS can significantly reduce friction.^16^ However, research on the utilization of PDMS in powder form as a lubricant additive has not yet been conducted, leaving a considerable research gap.

The aim was to utilize the advantageous properties of PDMS as a soft material while addressing wear issues associated with conventional hard particles by evaluating the performance of PDMS in powder form as a lubricant additive. By exploiting the unique properties of PDMS, including its elasticity, wear resistance, and friction-reducing capabilities of its microstructured form, we hypothesized that PDMS powder can enhance lubrication performance while mitigating the wear issues associated with traditional additives. This approach represents a novel strategy in the field of lubricant additives, and potentially offers a solution that combines improved lubrication and enhanced wear resistance.

To this end, we evaluated the performance of PDMS powder as a lubricant additive, considering the aspects of friction reduction, improvement in wear resistance, and enhancement of system efficiency. Furthermore, we analyzed the variations in the chemical composition and chemical bonding at various mixing ratios and evaluated the viscosity, microstructure, wettability, and lubrication properties under diverse conditions.

## 2 Materials and methods

### 2.1 Materials

304 stainless steel (304 SS, 20 mm × 20 mm × 1 mm) was used as the base substrate. A polydimethylsiloxane (PDMS) base and curing agent (Sylgard 184, DOW, Republic of Korea) were used to prepare powder additives. A bare lubricant solution (ZIC X7; SK Enmove Co., Ltd, Republic of Korea) was also prepared. The key properties of the base lubricant oil and the main characteristics of the PDMS powder used as a lubricant additive are summarized in Tables 1 and 2, respectively.^17–20^

**Table 1:** Key properties of the base lubricant oil

Key properties of base lubricant oil	
SAE viscosity	5W30
Density	0.85 g cm^−3^
Kinematic viscosity@40 °C	70.82
Kinematic viscosity@100 °C	12.02
Viscosity index	167
Flash point	224 °C

**Table 2:** Main characteristics of PDMS powder used as a lubricant additive

Main characteristics of PDMS	
Elastic modulus	0.74–2.61 MPa
Poisson's ratio	0.5
Friction coefficient	1.05–1.54
Wear rate	3.92–9.09 × 10^−7^ mm^3^ N^−1^ mm^−1^
Wettability	Hydrophobic, oleophilic

### 2.2 Lubricants

**Fig. 1:**
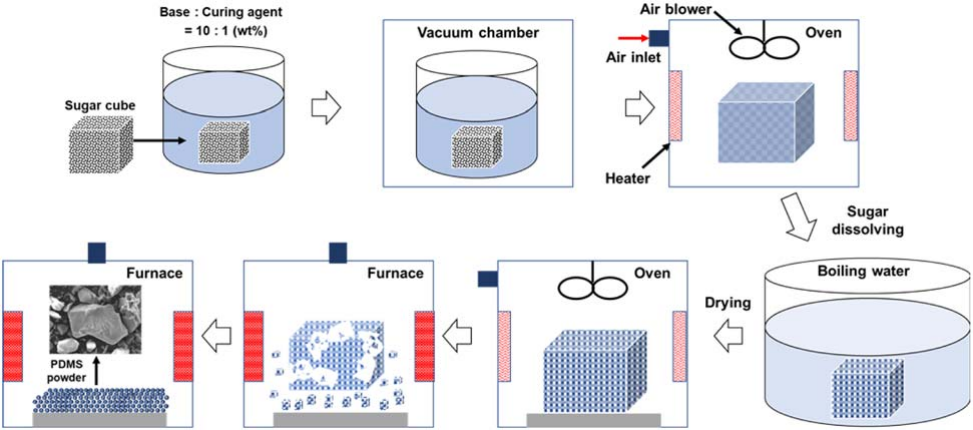
Schematic design of PDMS powder fabrication process.

The optimal synthesis conditions for the PDMS powder were determined based on previous research, with an emphasis on producing microparticles. To achieve a uniform heat distribution during the synthesis process, we utilized the sugar template method rather than plate formation. This method resulted in a porous, sponge-like structure that allowed for more uniform powder formation throughout the material, as opposed to the gradual formation observed in the plate structures. Fig. 1 illustrates the process of PDMS powder preparation. To begin the synthesis process, we first combined PDMS base and curing agent in a 10 : 1 weight ratio to create a PDMS mixture. The PDMS mixture was then poured over the sugar template and vacuum-treated for 1 h to remove the air bubbles. After curing at 70 °C for 3 h, the cured PDMS–sugar composite was ground and washed with hot deionized water (>90 °C) to remove the sugar, resulting in a porous PDMS structure. Finally, we heated this porous PDMS at 350 °C for 3 hours and then cooled it to room temperature to yield the PDMS powder. Our research showed that the particle size and shape of the powder depend significantly on the heating temperature and duration. As the temperature and heating time increased, the particles became more compact, with sizes ranging from tens to hundreds of nanometers. For the tribological evaluation, we employed 304 SS plates measuring 20 mm × 20 mm × 1 mm as the substrate material. Lubricants were prepared by incorporating varying concentrations of PDMS powder into the base lubricant at 0, 0.5, 1, 1.5, 2, and 2.5 wt%. To ensure initial dispersion, the mixture was stirred using a magnetic stirrer at 300 rpm for 1 h, followed by sonication for 20 min to achieve uniform dispersion and to break up any agglomerates.

### 2.3 Experiments

Various analytical and characterization methods have been employed to investigate the characteristics and lubrication performance of PDMS powder as a lubricant additive. Scanning electron microscopy (SEM; JSM-IT300, JEOL, Tokyo, Japan) was used to determine the size and morphology of the PDMS powder. Additionally, energy-dispersive X-ray spectroscopy (EDS) analysis was performed to determine the elemental composition of the PDMS powder, and elemental mapping was conducted to visualize the distribution of carbon (C), oxygen (O), and silicon (Si) within the powder particles. X-ray diffraction (XRD, EMPyrean, PANalytical, Malvern, UK) was employed to analyze the crystal structure of the PDMS powder, and Fourier-transform infrared spectroscopy (FTIR-ATR, Nicolet 6700, Thermo Scientific, Seoul, Korea) was used to quantitatively and qualitatively assess the chemical compositions of the bare lubricant solution and PDMS powder. Additionally, the viscosity of the lubricant according to the amount of PDMS powder added was measured using a viscometer (SV-10, A&D Co., Ltd, Tokyo, Japan), and the surface wettability of each lubricant was determined by measuring the oil droplet contact angle (OCA). OCA was measured by placing 10 μL of oil at five random locations on the surface of 304 SS and averaging the results. X-ray photoelectron spectroscopy (XPS, K-Alpha+, Thermo Scientific, USA) was conducted to investigate the chemical composition and bonding states of the PDMS powders. XPS measurements were performed using a spectrometer equipped with a monochromatic AlK_α_ X-ray source. Survey scans and high-resolution spectra of C 1s, O 1s, and Si 2p were acquired for both the bare and crosslinked PDMS powders.

**Fig. 2:**
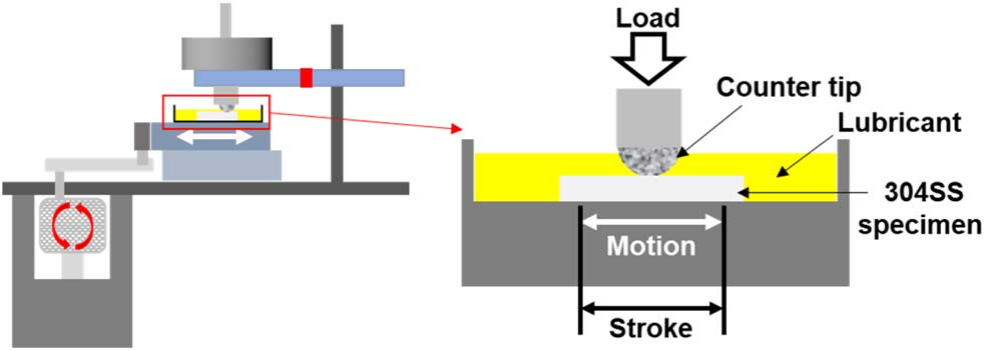
Schematic diagram of the tribotester.

A frictional analysis was conducted using a reciprocating sliding tribotester (RFW 160, NEOPLUS, Co., Ltd, Daejeon, Korea) to analyze the friction characteristics according to the lubricants at various concentrations of PDMS powder. The experimental setup for the friction testing is illustrated in Fig. 2. The friction test conditions are listed in Table 1. A 304 SS substrate was used, and a counter tip with a diameter of 1 mm made of 304 SS was employed. The sliding speed, normal load, and number of sliding cycles were set to 16 mm s^−1^, 100 mN, and 50 000 cycles, respectively. The wear track on the surface of the 304 SS substrate was analyzed using an optical microscope (DM750, Leica, Wetzlar, Germany) and a 2D profiler (SV-2100M4, Mitutoyo Korea Corporation, Gunpo, Korea), and the wear volume was calculated by measuring the wear width and wear depth. The wear rate is the ratio of the wear volume to the product of the normal load and total sliding distance.^19,21^ To ensure the reliability of the friction tests, the experiments were conducted at least five times under each condition and averaged.

## 3 Results and discussion

An investigation was conducted to evaluate the effect of PDMS powder on the friction and wear properties of the lubricating oil. Silicone rubber powder was successfully fabricated using a heat treatment technique.

**Fig. 3:**
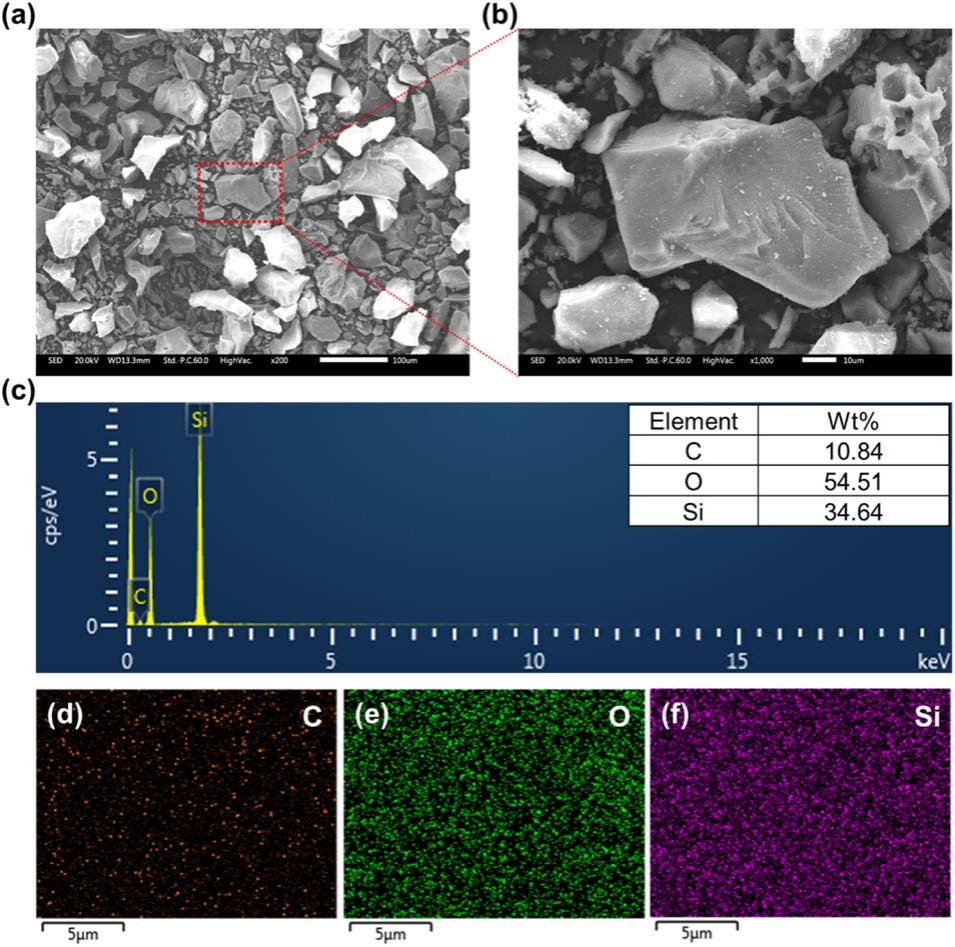
Characterization of PDMS powder: (a) low magnification SEM image, (b) high magnification SEM image, (c) EDS spectrum, elemental mapping images of (d) carbon, (e) oxygen, and (f) silicon within the powder particles.

**Fig. 4:**
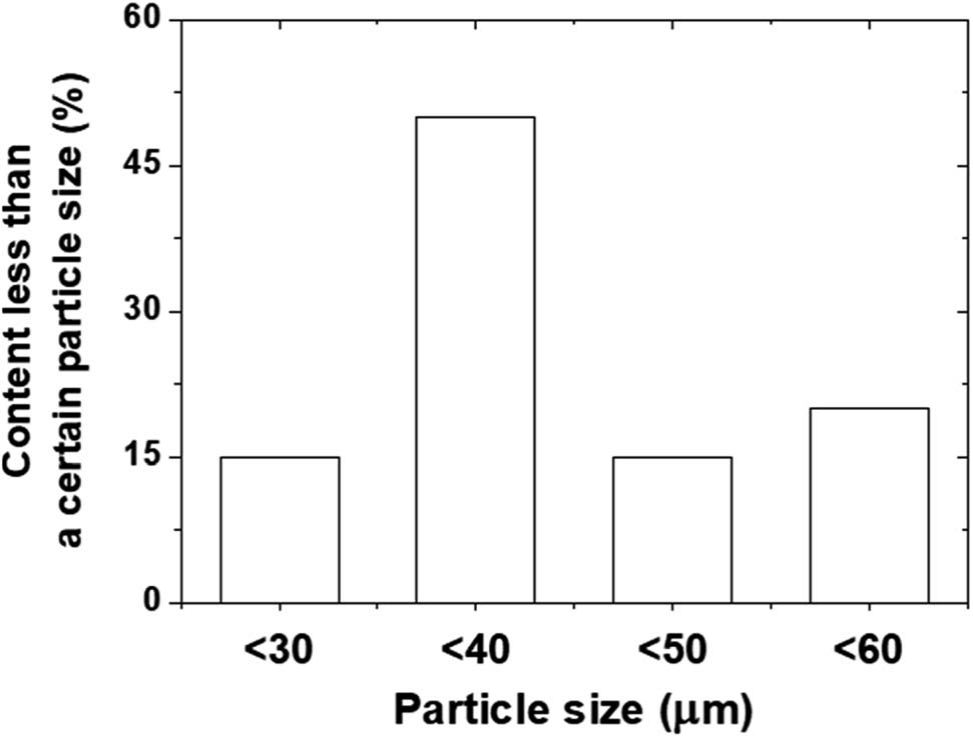
Particle distribution of PDMS powders.

Fig. 3 provides a detailed characterization of the PDMS powder. The low-magnification SEM image (Fig. 3(a)) shows the overall morphology and size distribution of the powder particles, while the high-magnification image (Fig. 3(b)) reveals the surface details of the individual particles. The EDS analysis (Fig. 3(c)) confirms the elemental composition of the PDMS powder, with carbon, oxygen, and silicon as the main constituents. The quantitative analysis shows that the powder consists of 10.84 wt% C, 54.51 wt% O, and 34.64 wt% Si, which is consistent with the expected composition of PDMS. The elemental mapping of C (Fig. 3(d)), O (Fig. 3(e)), and Si (Fig. 3(f)) demonstrated the uniform distribution of these elements throughout the powder particles, further confirming the homogeneity of the synthesized PDMS powder. The powder exhibited a wide particle size distribution, ranging from approximately 5 μm to 70 μm, with an average size of approximately 39 μm, as shown in Fig. 4. This particle size range is highly suitable for lubricant applications because smaller particles effectively fill surface voids, reduce direct contact between metal surfaces, and form protective films. Additionally, larger particles contribute to the load distribution and stress reduction, further enhancing the wear resistance.

**Fig. 5:**
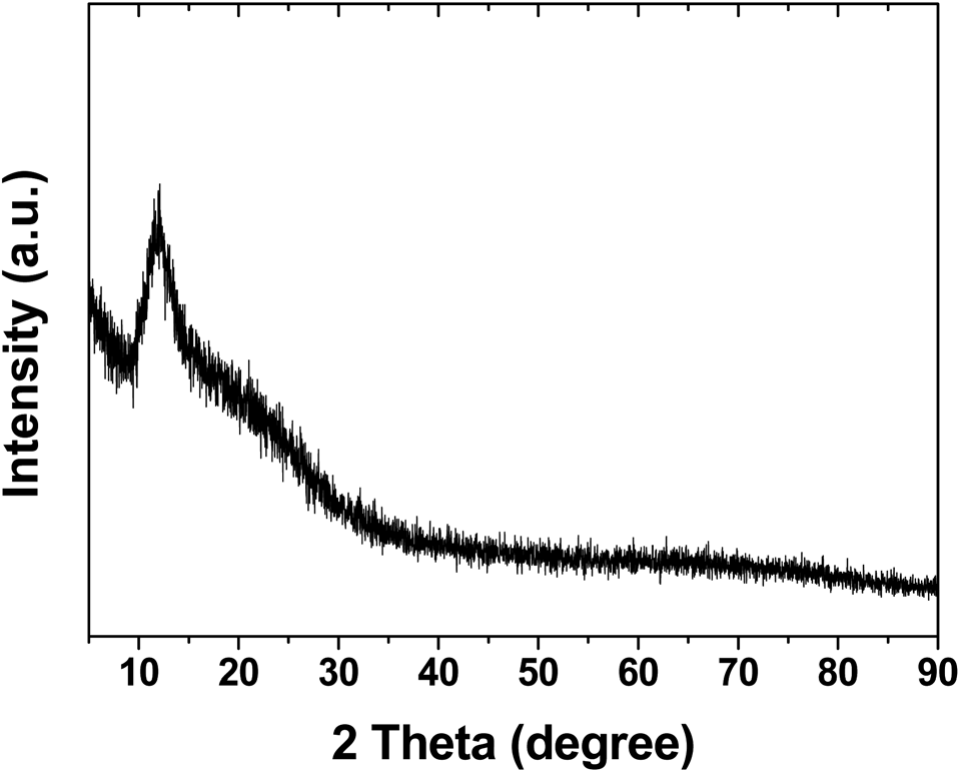
X-ray diffraction pattern of PDMS powder.

The X-ray diffraction (XRD) pattern of the PDMS powder is presented in Fig. 5. The XRD analysis revealed a characteristic peak at 11° for the PDMS powder, corresponding to its amorphous structure.^17^ This amorphous nature is a key factor contributing to the soft and deformable properties of the PDMS powder and plays a crucial role in its friction performance. The sharpness of the peak indicates high crystallinity, suggesting the effective crystallization of PDMS during the heat treatment process. The absence of other major peaks indicates that the PDMS powder consists of a single crystalline phase.^22^

**Fig. 6:**
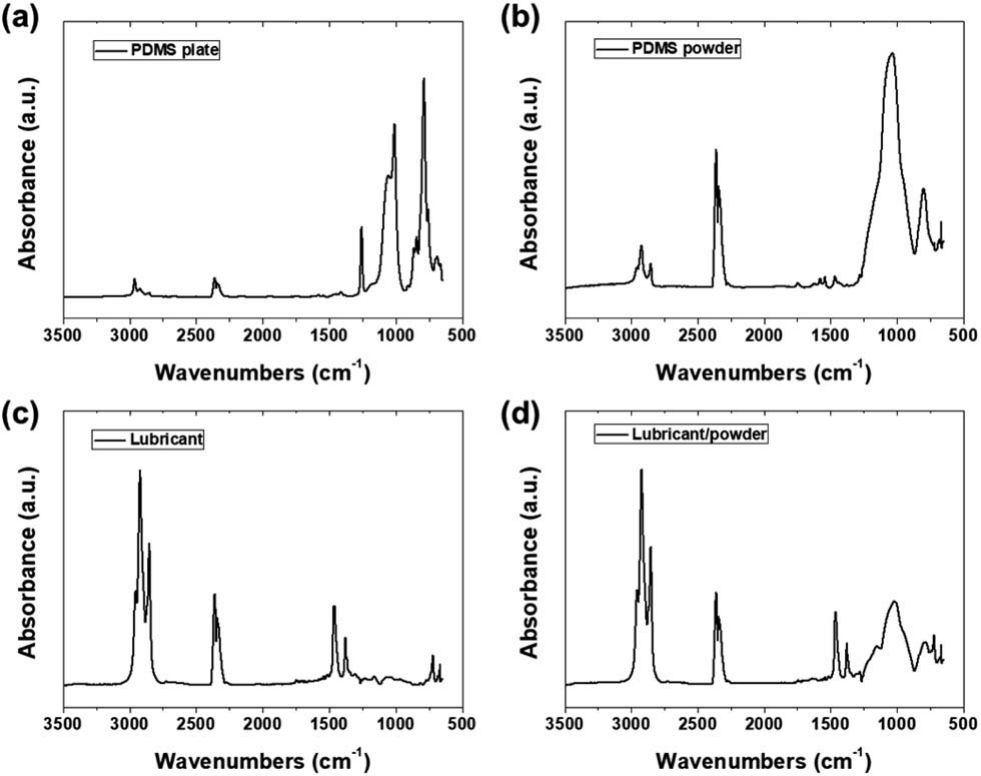
FTIR-ATR analysis of (a) PDMS plate, (b) PDMS powder, (c) lubricant, and (d) lubricant/PDMS powder.

As shown in Fig. 6, FTIR spectroscopy was used to study the molecular structures of the PDMS plate, powder, and lubricant. The FTIR spectrum confirmed the presence of the PDMS and PAO components and the successful dispersion of the PDMS powder within the bare lubricant solution. The FTIR spectrum of the PDMS plate exhibited characteristic peaks at 2962, 1257, 1010, 843, 788, 756, and 687 cm^−1^, corresponding to the stretching, rocking, and deformation vibrations of the C–H bonds of methyl groups, asymmetric and symmetric stretching vibrations of Si–O–Si bonds, and vibrations of Si–C bonds in the PDMS structure.^18,23^ These distinct peaks confirm the presence of the PDMS structure in the plate sample. For the PDMS powder, similar peaks to the PDMS plate were observed at 2922, 2851, 1541, 1031, and 799 cm^−1^, corresponding to C–H stretching vibrations of methyl groups, bending vibrations of Si–CH_3_ groups, asymmetric stretching of Si–O–Si bonds, and symmetric stretching of Si–C bonds, respectively.^24,25^ The peaks for the PDMS powder showed slight shifts and additional peaks compared to the PDMS plate, indicating changes in the molecular environment and potential interactions during the powder fabrication process, such as heat treatment. The spectrum of the PAO-based lubricant exhibited peaks at 2954, 2852, 1463, 1377, 721, and 668 cm^−1^, representing the stretching, bending, rocking, and deformation vibrations of the C–H bonds.^26,27^ These peaks align with the chemical composition of lubricants composed of long-chain hydrocarbon molecules. Notably, the FTIR spectrum of the PDMS powder-lubricant composite displayed a combination of peaks from the PDMS powder and the PAO-based lubricant. The peaks at 2954, 1463, 1377, 721, and 668 cm^−1^ correspond to vibrations of alkane/alkyl groups in the PAO lubricant, while the peaks at 2922, 1011, and 787 cm^−1^ were attributed to the vibrations of methyl groups and the Si–O–Si backbone in PDMS powder. The peak at 2825 cm^−1^ was associated with the stretching vibrations of the C–H bonds of the methyl groups and potentially resulted from interactions between the PDMS powder and lubricant matrix. The presence of peaks related to both PDMS and the bare lubricant solution in the composite spectrum confirms the successful dispersion of the PDMS powder in the lubricant. The observed peaks and their values support the mechanisms and interactions between the PDMS powder and bare lubricant solution, contributing to lubrication film formation, wear resistance improvement, and overall enhancement in friction performance.

**Fig. 7:**
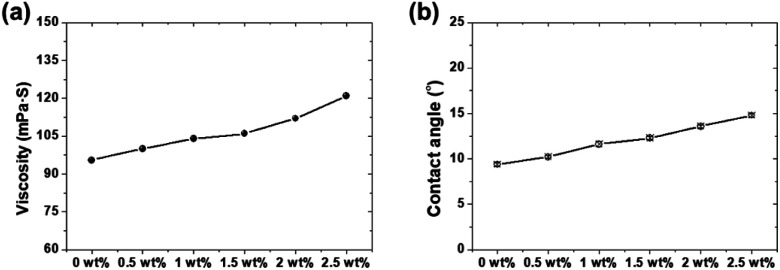
(a) Viscosity of the lubricant and (b) contact angles on 304 SS substrates as a function of PDMS powder concentration.

Fig. 7 shows the viscosity and OCA results of the lubricant according to the PDMS powder concentration. The viscosities of the lubricants were 95.5, 100, 104, 106, 112, and 121 mPa S for 0, 0.5, 1, 1.5, 2, and 2.5 wt%, respectively. The observed increase in viscosity may be attributed to the rheological behavior of polymer-based silicone rubber powder particles. Under shear stress, these deformable particles align and entangle, increasing the resistance to fluid flow and consequently elevating the lubricant viscosity.^28^ This viscosity enhancement significantly influences lubrication film formation. Additionally, as depicted in Fig. 7(b), the contact angle of each lubricant increased with increasing PDMS powder concentration, measuring 9.34, 10.21, 11.63, 12.29, 13.59, and 14.8° for 0, 0.5, 1, 1.5, 2, and 2.5 wt%, respectively. This increase in the contact angle with increasing PDMS powder content indicates enhanced wettability and wear resistance.^29^ The hydrophobic properties of the PDMS powder contribute to surface wettability, facilitating better spreading and adhesion of the lubricant on the surface.^30^ This improved wetting promotes the formation of a more stable and protective lubrication film, which is expected to enhance wear resistance.

**Fig. 8:**
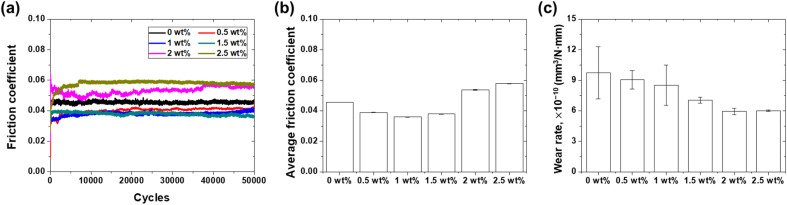
Friction and wear characteristics of lubricants as a function of PDMS powder concentration: (a) friction coefficient history, (b) average friction coefficient, and (c) wear rate.

Fig. 8 illustrates the friction and wear characteristics of each lubricant with respect to PDMS powder concentration. As shown in Fig. 8(a), the friction coefficient started at 0.045 for 0 wt% and remained constant. Conversely, with the addition of PDMS powder, the friction coefficient exhibited a slightly increasing trend with increasing sliding cycles; however, significant differences in the friction coefficient were not observed during 50 000 cycles of sliding friction. Fig. 8(b) shows the average friction coefficient of each lubricant according to the PDMS powder concentration. While 0 wt% exhibited friction coefficient of 0.05, 0.5, 1, 1.5, 2, and 2.5 wt% exhibited friction coefficients of 0.04, 0.04, 0.04, 0.05, and 0.06, respectively. The friction coefficient remained relatively stable with increasing PDMS powder concentration, indicating that the powder did not adversely affect the friction behavior of the lubricant. This is attributed to the deformable nature of the powder particles, which easily deform under contact pressure, minimizing direct contact between metal surfaces and maintaining the lubrication film.^31^ The observed friction behavior is attributed to surface interactions and lubrication film formation mechanisms, where smooth PDMS powder particles deform easily under sliding contact and attach to the contacting surface to reduce friction by forming a tribofilm.^32^ Furthermore, the motion of larger powder particles contributes to stress distribution mechanisms, further minimizing frictional losses.^33^ Thus, the observed friction behavior in lubricants with added PDMS powder stems from the inherent physicochemical properties and the associated wettability and mechanical behavior of the powder. PDMS, a silicone-based polymer, has an amorphous structure with low elasticity, high deformation, and high oil affinity.^34^ In terms of the friction behavior, these characteristics indicate that powder particles uniformly dispersed in the lubricant under contact stress can deform easily. This deformation minimizes direct contact between the metal surfaces, and the powder particles support the load, reduce friction, and ensure stable friction behavior.

Fig. 8(c) shows the wear rates of the 304 SS substrate for 50 000 sliding cycles under lubrication conditions with varying PDMS powder concentrations. The wear rate for 0 wt% was 9.74 × 10^−10^ mm^3^ N^−1^ mm^−1^, while for 0.5, 1, 1.5, 2, and 2.5 wt%, it was 9.05, 8.5, 7.03, 5.95, and 6 × 10^−10^ mm^3^ N^−1^ mm^−1^, respectively. In particular, the wear rate decreased with increasing powder concentration, reaching a minimum of 2 wt%. This improvement in wear resistance can be attributed to the excellent elasticity and recovery performance of the PDMS powder, as well as the combined effect of lubrication film formation. The soft and deformable nature of PDMS powder particles plays a crucial role in reducing wear. When contact stress is applied, the particles deform easily, effectively reducing local pressure and preventing abrasive wear.^35^ This deformation mechanism is promoted by the amorphous structure and low elasticity of PDMS, which allows efficient energy dissipation and stress distribution upon contact. The smooth motion of lubricant-friendly powder particles can contribute to the observed wear resistance through lubrication effects. As the larger particles slide between the contacting surfaces, they act as surface protective films, thereby reducing direct contact. This lubrication effect is facilitated by the shape of the powder particles, which easily reduces frictional forces under shear stress. Additionally, the PDMS powder particles attach to the contacting surface, forming a thin tribofilm that protects the substrate from contact and wear. Specifically, larger PDMS particles (approximately 40–70 μm) in size distribution are effective in load distribution owing to their larger contact area. These particles act as microscopic cushions, dispersing the pressure applied to the contact area over a wider region.^36^ Simultaneously, due of the elastic properties of PDMS, these particles possess the ability to deform under load and recover their original shape, providing continuous stress relief even under repetitive loading conditions. Furthermore, these larger particles function as bearings within the lubricant film. They roll between relatively moving surfaces, thereby reducing friction while preventing direct contact between the two surfaces. This mechanism plays a crucial role, particularly under boundary lubrication conditions, significantly reducing wear. Additionally, the larger PDMS particles effectively maintained the thickness of the lubricant film. This enhanced the hydrodynamic lubrication effect, leading to a reduction in the friction coefficient and wear. Consequently, the synergistic effect of these various mechanisms, particularly the addition of larger PDMS particles, significantly improved the wear resistance of the lubricant.

**Fig. 9:**
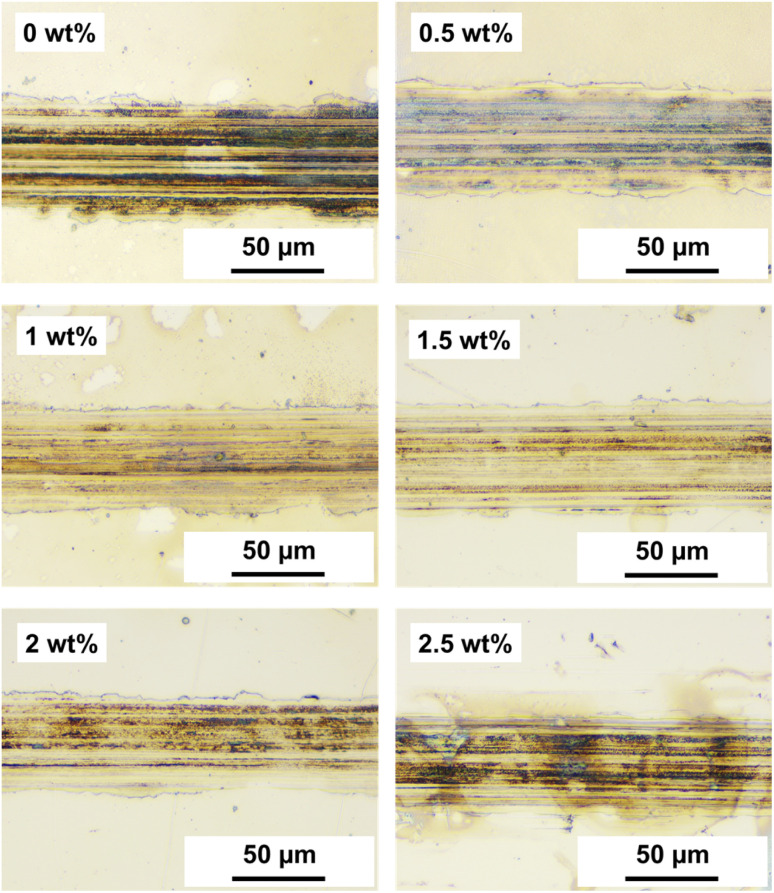
Optical microscope images of wear tracks on 304 SS substrate as a function of PDMS powder concentration.

As depicted in Fig. 9, optical microscopy analysis of the wear tracks provided visual evidence supporting the trends in friction behavior. Both 0 wt% and 0.5 wt% exhibited rough surface topography across the entire wear track, showing attachment of worn metal and PDMS powder particles. Additionally, a relatively wide wear width was formed with numerous scratch patterns across the surface. In contrast, the surfaces of the 304 SS substrates under lubrication conditions containing PDMS powder from 1 to 2 wt% showed relatively narrow wear widths and minimized wear particle attachment, corroborating the low wear rates observed in Fig. 8(c). The deformable nature of the powder particles likely contributed to the formation of a protective film, which protected the substrate surface from wear.^37^ However, at the highest concentration of 2.5 wt%, a slight increase in the wear rate was observed, with the surface appearing rougher in the optical microscopy images. This could be due to the aggregation and excessive accumulation of PDMS powder particles on the contacting surface, leading to destruction of the tribofilm and localized wear. Exceeding a certain threshold concentration of powder may induce particle aggregation owing to uneven dispersion within the lubricant matrix, leading to wear.^38^

**Fig. 10:**
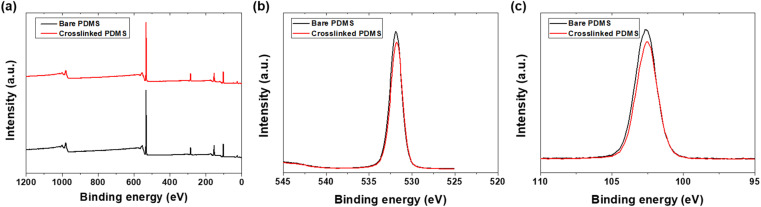
XPS analysis of bare PDMS and crosslinked PDMS powders: (a) survey spectra, (b) O 1s spectra, and (c) Si 2p spectra.

To evaluate the friction and wear performance comprehensively, a PDMS powder concentration of 1 wt% was identified as the optimum condition, exhibiting a favorable combination of low friction and wear rates. At this concentration, the PDMS powder particles were sufficiently dispersed within the lubricant matrix, facilitating effective deformation and tribofilm formation mechanisms, while minimizing aggregation and wear potential. To further enhance the wear resistance and durability of the lubricant blend, a polydimethylsiloxane (PDMS) curing agent was added. The addition of a curing agent can crosslink and strengthen the PDMS powder particles, thereby enhancing their resistance to contact loads. This crosslinking process occurs through the formation of covalent bonds between PDMS chains, resulting in the formation of a more robust and wear-resistant tribofilm.^39^ To further investigate the chemical composition and bonding states of the PDMS powders, XPS analysis was performed on both the bare and crosslinked PDMS. Fig. 10 presents the XPS survey spectra and high-resolution spectra of C 1s, O 1s, and Si 2p for both the powders. The survey spectra confirmed the presence of carbon, oxygen, and silicon in both powders.

Analysis of the high-resolution spectra revealed subtle but significant differences between the bare and crosslinked PDMS powder. The O 1s peaks for both powders were observed at binding energies of approximately 532 eV, which is characteristic of Si–O bonds in PDMS.^40^ However, the crosslinked powder showed a slight shift to a lower binding energy (531.96 eV) compared to the bare powder (532.05 eV). This shift suggests a change in the chemical environment of oxygen atoms, potentially indicating an increase in Si–O–Si crosslinking.^41^ The Si 2p spectra also exhibited a similar trend, with the crosslinked powder showing a peak at a slightly lower binding energy (102.83 eV) compared to the bare powder (102.92 eV). This shift is consistent with increased Si–O–Si bonding, further supporting enhanced crosslinking in the crosslinked powder.^42^ Quantitative analysis of the XPS data revealed changes in the atomic percentages of the elements. For the crosslinked powder, the O/Si ratio increased from 1.91 in the bare powder to 1.94 in the crosslinked powder, indicating a higher proportion of oxygen relative to silicon. This increase was consistent with the formation of additional Si–O–Si crosslinks in the crosslinked powder. Moreover, the C 1s spectra showed a slight increase in the proportion of C–O bonds in the crosslinked powder, suggesting the incorporation of additional oxygen-containing groups during the modification process. This change in the carbon bonding environment further supports the occurrence of structural modifications that enhance the crosslinking. The XPS results provided evidence of increased crosslinking in the crosslinked PDMS powder. The observed shifts in binding energies, changes in elemental ratios, and alterations in bonding environments all indicate a higher degree of Si–O–Si network formation in the crosslinked powder. This enhanced crosslinking was expected to improve the mechanical properties and wear resistance of the crosslinked powder when used as a lubricant additive.

**Fig. 11:**
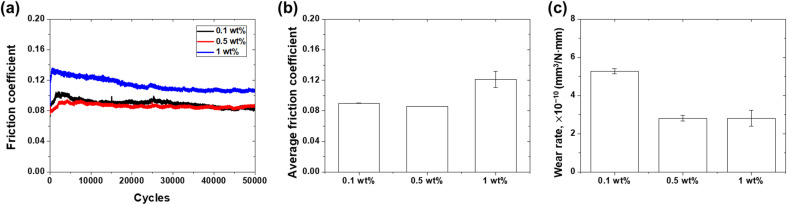
Friction and wear characteristics of lubricants as a function of curing agent concentration: (a) friction coefficient history, (b) average friction coefficient, and (c) wear rate.

**Fig. 12:**
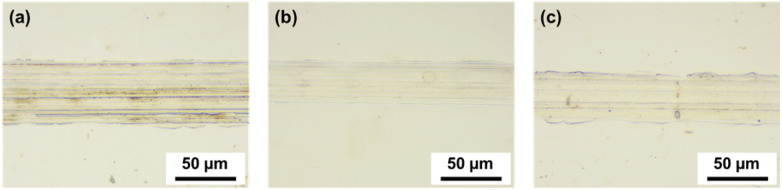
Optical microscope images of wear tracks on 304 SS substrates as a function of curing agent concentration: (a) 0.1 wt%, (b) 0.5 wt%, and (c) 1 wt%.

Fig. 11(a) shows the friction coefficient of the lubricant as a function of the curing agent concentration. In the case of the lubricant with a 0.1 wt% curing agent, the friction coefficient started at a high value of above 0.12 and gradually decreased with increasing sliding cycles. Conversely, for lubricants with 0.1 wt% and 0.5 wt% curing agents, the friction coefficient initially increased slightly but then remained constant. As shown in Fig. 11(b), the average friction coefficients for 0.1, 0.5, and 1 wt% were 0.09, 0.09, and 0.12, respectively. The friction coefficient exhibited a relatively large value at 1 wt%, suggesting that the friction coefficient may increase with increasing curing agent concentration. Conversely, as shown in Fig. 11(c), the wear rates for 0.1 wt%, 0.5 wt%, and 1 wt% were 5.27, 2.81, and 2.81 × 10^−10^ mm^3^ N^−1^ mm^−1^, respectively, showing a decreasing trend with curing agent concentrations of 0.5 wt%. Fig. 12 shows the influence of the curing agent on the wear resistance through an optical microscopy analysis of the wear tracks. The lowest concentration of 0.1 wt% exhibited the widest wear width and scratch patterns, while 0.5 wt% and 1 wt% showed almost no scratches and relatively small wear widths. The addition of a curing agent can affect the surface interactions and tribofilm formation mechanisms. The curing agent strengthened the mechanical properties of the PDMS powder particles, enhanced the adhesion and affinity with the contact surface, and promoted more stable and durable tribofilm formation. Additionally, the crosslinked structure of the cured PDMS particles improves their resistance to deformation and wear, thereby enhancing the lifespan and performance of the lubricant under operating conditions. The concentration of the curing agent should be optimized to strike a balance between improved wear resistance and friction behavior. Excessive crosslinking may lead to negative changes in rheological behavior, surface interactions, and compatibility with the lubricant, ultimately impairing the overall friction performance.^43^

**Fig. 13:**
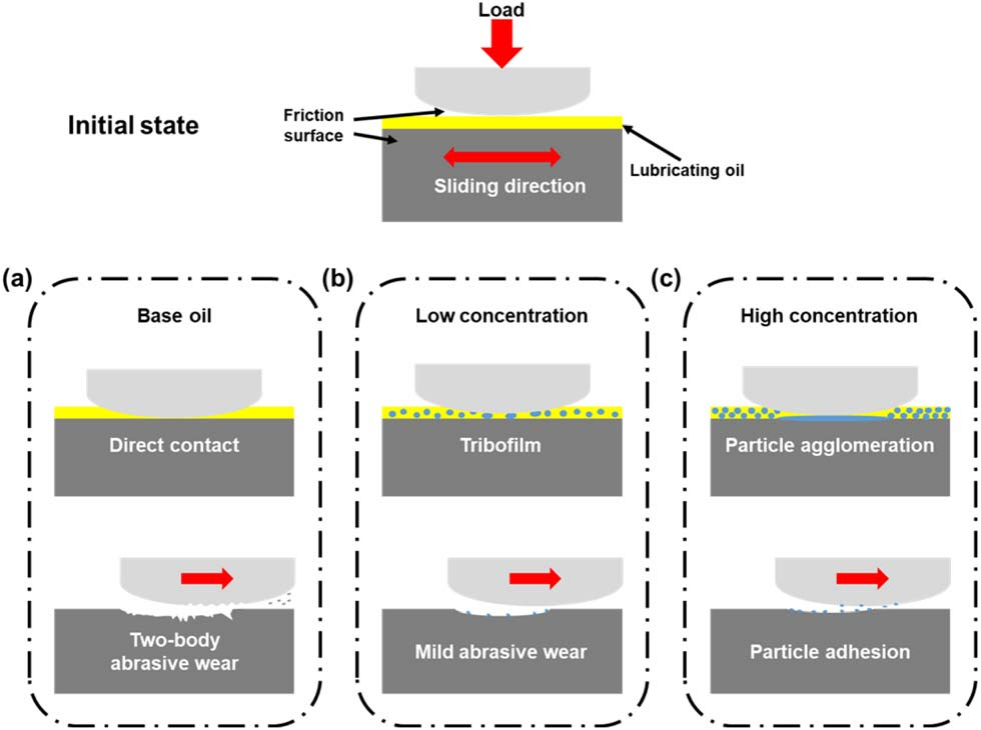
Schematic design of the lubrication mechanism as a function of PDMS powder concentration: (a) 0 wt%, (b) 1–1.5 wt%, and (c) 2–2.5 wt%.

The performance evaluation presented in this study provides insights into the mechanisms that determine the friction and wear behavior of lubricants containing PDMS powder. As shown in Fig. 13, the soft and deformable nature of PDMS powder has emerged as a key factor contributing to enhanced lubrication and wear resistance by interacting with the lubricant surface and forming a stable tribofilm.^44^ The amorphous structure and low elasticity modulus of the PDMS powder particles facilitated effective deformation under contact stress, reducing the local contact pressure and minimizing surface wear. Additionally, the chemical properties and surface wettability of the PDMS powder contributed to the tribofilm formation. The hydrophobic nature of the powder particles was demonstrated by the increased contact angle and enhanced surface wetting and adhesion, facilitating the formation of a stable tribofilm that protects the metal substrate from wear.^45^ Optimization of the PDMS powder concentration plays a crucial role in improving the friction performance. At low concentrations, excellent friction and wear characteristics were observed owing to the effective interactions, such as sufficient dispersion, deformation, and lubricating film formation. Conversely, excessive concentrations led to particle aggregation and destruction, resulting in a slight increase in the wear rate. The addition of a PDMS curing agent offers a solution to enhance performance. By crosslinking and bonding the PDMS particles, the curing agent improves the mechanical strength, potentially further enhancing the wear resistance and durability.^46^

## 4 Conclusions

This study highlights the potential of PDMS powder as a promising additive for lubricants, offering both lubricating and antiwear properties. Silicone rubber powder, synthesized *via* sugar templating and heat treatment, exhibited a particle size distribution ranging from 5 to 70 μm, with an average size of approximately 39 μm. XRD analysis confirmed the amorphous structure of PDMS, imparting soft and deformable characteristics that are crucial for the friction performance. FTIR analysis validated the presence of PDMS and PAO components, along with the successful dispersion of the PDMS powder within the lubricating oil. XPS analysis demonstrated a notable increase in crosslinking within the crosslinked PDMS powder. This is apparent from the variations in the binding energies and alterations in the elemental ratios. The addition of silicone rubber powder increased the viscosity of the lubricating oil from 95.5 mPa S at 0 wt% to 121 mPa S at 2.5 wt%, while the contact angle rose from 9.34° to 14.8°, indicating improved load-carrying capacity. Friction tests revealed that PDMS powder did not adversely affect the friction behavior, maintaining an average friction coefficient of 0.04–0.06 within the 0.5–2.5 wt% range. Meanwhile, the wear rate decreased with increasing PDMS powder concentration, reaching a minimum of 5.95 × 10^−10^ mm^3^ N^−1^ mm^−1^ at 2 wt%, which is attributed to particle deformation, stress absorption/dispersion effects, and lubricating film formation. Optical microscopy analysis confirmed narrow wear widths and minimal wear particle adhesion in the specimens with 1 wt% and 2 wt% concentrations. The addition of the PDMS curing agent in the range of 0.1–1 wt% resulted in a decreased wear rate, reaching 2.81 × 10^−10^ mm^3^ N^−1^ mm^−1^ at concentrations exceeding 0.5 wt%. The curing agent enhanced the mechanical properties of the PDMS powder particles through cross-linking, improving surface adhesion and forming a more stable and durable lubricating film. In conclusion, this study suggests the potential to enhance lubrication performance through PDMS powder concentration optimization and curing agent addition. The soft and deformable characteristics of powder particles, along with their interaction with surfaces, have been identified as key factors for improving the lubrication and anti-wear properties. In particular, at low concentrations of 1–2 wt%, the effective dispersion, deformation, and lubricating film formation mechanisms of the powder particles demonstrated superior friction and wear properties. These findings indicate the potential of PDMS powder in various industries, where lubrication performance and component lifespan improvement are critical lubrication applications.

Although this study provides valuable insights into the potential of PDMS powder as a lubricant additive, there are several areas that warrant further investigation. Future research should explore the behavior of PDMS powder additives under various temperature conditions Additionally, the potential agglomeration of PDMS particles, especially under high temperatures or prolonged use, requires examination to understand its impact on long-term stability and tribological performance. Finally, although this study demonstrated promising results with the addition of a curing agent, further research into optimizing the curing process and investigating its long-term effects on lubricant performance would be beneficial. These future directions will contribute to a more comprehensive understanding of PDMS powder as a lubricant additive and potentially lead to its optimized application in various industrial settings.

## Data availability

The datasets generated and analyzed during the current study are available within the manuscript itself. All relevant data are presented in the form of figures, tables, and supplementary information accompanying this article.

## Author contributions

Sung-Jun Lee: conceptualization, methodology, software, validation, formal analysis, investigation, data curation, writing – original draft, writing – review & editing, visualization. Dawit Zenebe Segu: commented on manuscript, resources, validation. Chang-Lae Kim: conceptualization, methodology, resources, writing – review & editing, supervision, project administration.

## Conflicts of interest

There are no conflicts to declare.
